# Assessment of Fraud Deterrence and Detection Procedures Used in a Web-Based Survey Study With Adult Black Cisgender Women: Description of Lessons Learned and Recommendations

**DOI:** 10.2196/59955

**Published:** 2025-03-12

**Authors:** Amber I Sophus, Jason W Mitchell

**Affiliations:** 1 Department of Health Promotion and Disease Prevention Robert Stempel College of Public Health & Social Work Florida International University Miami, FL United States

**Keywords:** Black women, HIV, fraud deterrence, fraud detection, web-based research, online research, data integrity, data collection, survey

## Abstract

**Background:**

Online research studies enable engagement with more Black cisgender women in health-related research. However, fraudulent data collection responses in online studies raise important concerns about data integrity, particularly when incentives are involved.

**Objective:**

The purpose of this study was to assess the strengths and limitations of fraud deterrence and detection procedures implemented in an incentivized, cross-sectional, online study about HIV prevention and sexual health with Black cisgender women living in Texas.

**Methods:**

Data for this study came from a cross-sectional web-based survey that examined factors associated with potential pre-exposure prophylaxis use among a convenience sample of adult Black cisgender women from 3 metropolitan areas in Texas. Each eligibility screener and associated survey entry was evaluated using 4 fraud deterrence features and 7 fraud detection benchmarks with corresponding decision rules.

**Results:**

A total of 5862 respondents provided consent and initiated the eligibility screener, of whom 2150 (36.68%) were ineligible for not meeting the inclusion criteria, and 131 (2.23%) completed less than 80% of the survey and were removed from further consideration. Other entries were removed for not passing level 1 fraud deterrent safeguards: duplicate entries with the same IP address (388/5862, 6.62%), same telephone number (69/5862, 1.18%), same email address (114/5862, 1.94%), and same telephone number and email address (17/5862, 0.29%). Of the remaining 2993 entries, 1652 entries were removed for not passing the first 2 items of the level 2 fraud detection benchmarks: screeners and surveys with latitude and longitude coordinates outside of the United States (347/2993, 11.59%) and survey completion time of less than 10 minutes (1305/2993, 43.6%). Of the remaining 1341 entries, 130 (9.69%) passed all 5 of the remaining level 2 data validation benchmarks, and 763 (56.89%) entries were removed due to passing less than 3. An additional 33.4% (423/1341) entries were removed after passing 4 of the 5 remaining validation benchmarks, being contacted to verify survey information, and not providing legitimate contact information or being unable to confirm personal information. The final enrolled sample in this online study consisted of 155 respondents who provided consent, were deemed eligible, and passed fraud deterrence features and fraud detection benchmarks. In this paper, we discuss the lessons learned and provide recommendations for leveraging available features in survey software programs to help deter bots and enhance fraud detection procedures beyond relying on survey software options.

**Conclusions:**

Effectively identifying fraudulent responses in online surveys is an ongoing challenge. The data validation approach used in this study establishes a robust protocol for identifying genuine participants, thereby contributing to the removal of false data from study findings. By sharing experiences and implementing thorough fraud deterrence and detection protocols, researchers can maintain data validity and contribute to best practices in web-based research.

## Introduction

### Background

In the past 2 decades, there has been a rise in online health-related research studies. Online research provides a practical approach to recruitment and data collection, including electronic dexterity, participant anonymity, speed, reduced error, easy and remote participation [1], cost efficiency, and the potential to recruit larger study samples [2,3]. While online research has expanded recruitment opportunities, Black/African Americans remain underrepresented in health-related research studies [4-9], particularly Black women [7-9] who are considered a “hard-to-reach” population [9,10].

Online research studies offer one way to engage more Black cisgender women in health-related research. However, conducting research online, particularly when incentives are involved, warrants careful attention. For instance, respondents may misrepresent themselves to improve their likelihood of meeting the study eligibility criteria to obtain the incentive [11,12]. The lack of face-to-face interaction (eg, video) restricts researchers’ ability to verify whether the data come from real individuals [13,14]. Other challenges associated with conducting online research include the presence of bots (ie, computer program software designed to automatically fill out survey questions with preprogrammed responses), the ability to bypass IP address restrictions, and respondent misrepresentation (ie, ineligible participants providing inaccurate information to gain entry into the study) [2,13,14]. Although online recruitment (ie, through social media) has been successful in attracting underrepresented populations in health-related research studies involving incentives [15-18], the use of a standard web link for online recruitment purposes may exacerbate the potential for fraudulent responses and threats to data integrity [19-22]. In response to these challenges, researchers have provided recommendations for how best to screen for fraudulent survey entries and handle potentially invalid responses [11,12,22-26].

Teitcher et al [12] recommends checking for inconsistent survey responses, using a CAPTCHA, collecting paradata to examine how individuals are responding to survey questions, tracking personal information (eg, email, home address, or telephone number), checking for encrypted IP addresses or multiple survey entries from the same IP address, enabling cookies to prevent multiple survey completion attempts, and including an interview to determine whether an individual has already participated or is being dishonest on responses. Other researchers have published similar recommendations [2,23,27,28]. In addition to these recommendations, researchers are encouraged to develop a system of decision rules to detect and handle invalid and fraudulent research data [11-13,25]. Pozzar et al [21] investigated threats to their sample validity and data integrity after online social media recruitment of health research participants to complete an online survey. Although the authors used a data collection platform with fraud prevention features, collected verifiable information, and included open-ended items to identify those who provided false information regarding their eligibility criteria, many respondents still bypassed the validity and data integrity measures [21]. Dewitt et al [29] applied similar participant validity procedures in a small internet health survey among a sample of gay and bisexual survivors of prostate cancer. However, the authors discovered that some invalid entries bypassed their validation protocol, although they passed checks for nonduplicate IP addresses, valid zip codes, and reCAPTCHA verification [29]. These findings underscore the importance of ongoing vigilance and periodic reassessment of validation strategies throughout the recruitment phase of a study to enhance the overall effectiveness of fraud prevention strategies. Furthermore, the tactics used to deceive or manipulate online study eligibility and enrollment systems are continually evolving and, as such, call for new insights and lessons learned to help improve rigor and data integrity.

### Purpose

This analysis builds on extant literature by assessing the strengths and limitations of fraud deterrence and detection procedures that were implemented in an incentivized, cross-sectional, online study about HIV prevention and sexual health with a convenience sample of Black cisgender women living in Texas, United States. We share the lessons learned from implementing this study and discuss different strategies that are available to help improve the likelihood of collecting valid data from legitimate research participants.

## Methods

This study adhered to the STROBE (Strengthening the Reporting of Observational Studies in Epidemiology) reporting guidelines [30].

### Recruitment

Between December 2020 and January 2022, we recruited Black cisgender women from Houston, San Antonio, and Dallas in Texas to complete a 1-time online study survey about HIV prevention and women’s sexual health, with a primary focus on pre-exposure prophylaxis [31]. Participants were recruited using online advertisements and print flyers that contained information about the study and a web link to access the online consent form and eligibility screener. Online advertisements were placed on Facebook and Instagram (clickable web link embedded), whereas printed flyers (with a QR code and web link) were placed in locations (eg, coffee shops, restaurants, grocery stores, and gyms) in Houston, Dallas, and San Antonio frequented by Black women. Snowball sampling was also used to recruit participants. All recruitment materials contained information about the study and a method (eg, QR code or direct web link) to access the online consent form and eligibility screener [32].

### Procedures

Irrespective of the recruitment method, all interested individuals who clicked the provided web link were taken to the study landing page housed in Qualtrics (Qualtrics International Inc), a Health Insurance Portability and Accountability Act (HIPAA) compliant survey program. The landing page provided information about the study, the electronic consent form, and the online eligibility screener. The electronic consent form provided detailed information about the survey study, such as its purpose and participation process (including time commitment), incentives, privacy and confidentiality, risks and benefits, and contact details for the primary investigator and institutional review board. Respondents who consented to participate were immediately routed to the eligibility screener. Once consented and eligible, individuals were prompted to provide their email address for incentive distribution before being automatically granted access to complete the 30-minute online study survey. Individuals were also instructed to provide their telephone number if they consented to participate in a possible 1-time, online interview (if purposively selected). In addition, individuals could provide their contact information to be contacted for future research opportunities.

Consented and eligible individuals who completed at least 80% of the study survey were enrolled in the parent study once their data were evaluated and had passed the fraud deterrence and detection protocols (described later in the Methods section). Individuals who were ineligible were thanked for their interest in the study and provided with relevant resources related to HIV prevention.

### Eligibility Criteria

For study inclusion, participants had to meet all of the following eligibility criteria through self-report: (1) be at least 18 years of age; (2) self-identify as a cisgender woman; (3) self-identify as Black (ie, African American or Caribbean American [eg, Haitian American]); (4) have an HIV-negative or unknown serostatus; (5) live in or near (within 25 miles [approximately 40 km]) Houston, San Antonio, or Dallas in Texas; (6) have engaged in at least 1 HIV risk behavior within the past 6 months (ie, unprotected vaginal or anal sex with a male partner, injection drug use, or sex exchange) or have been diagnosed with a sexually transmitted infection (STI); and (7) be fluent in English. Individuals who did not meet all 7 eligibility criteria were excluded.

### Application of the First Level of Fraud Deterrent Protocols: Lesson 1

To help deter the collection of invalid data [11,23,27,33-37], 4 fraud prevention safeguards—built-in features available in Qualtrics—were selected for use in the online eligibility screener and study survey before recruitment began. The 4 fraud prevention safeguards are CAPTCHA verification, prevent indexing, prevent ballot box stuffing (ie, duplicate entries), and bot detection. Descriptions of the 4 fraud prevention safeguards are provided in Textbox 1.

**Descriptions of the level 1 fraud deterrent protocol, which included 4 fraud prevention safeguards used in the Qualtrics eligibility screener for all respondents.**

**CAPTCHA**

CAPTCHA requires a respondent to successfully pass a task or challenge (eg, select all squares containing fire hydrants in the image) to gain access to the next web page (eg, survey)

**Prevent indexing**

A tool that prevents bots or software from finding the survey within web search engines

**Prevent ballot box stuffing (currently called “Prevent Multiple Submissions”)**

A tool that places a cookie on the individual’s browser (cookies are small data files generated by a web server and sent to a web browser; they track user activity, help websites recognize returning users, and improve the browsing experience); if the same respondent returns using the same browser on the same device, without having cleared their cookies, they are flagged as a duplicate

**Bot detection**

A tool that assesses the likelihood of a response being from a bot or a human by assigning a probability score based on interactions with invisible Google reCAPTCHA technology embedded in the survey

Soon after recruitment began, there was a huge influx of data entries—1498 within the first 3 days. This unusual activity prompted us to pause the study (ie, recruitment as well as screener and survey) to evaluate whether the screener and study survey entries were valid. During this review, the safeguard options presented in Textbox 1 were thoroughly examined to determine whether there were limitations to using these 4 features.

We learned that the prevent ballot box stuffing option in Qualtrics does not fully prevent duplicate entries. Respondents could still access the survey by switching browsers or devices, even when this option was enabled. In addition, Qualtrics does not prevent duplicate entries based on additional criteria such as name, telephone number, email address, or IP address. Of the 1498 entries, approximately half (n=769, 51.3%) were linked to an entry that shared an IP address with at least 1 other survey entry. Several other patterns were noted while evaluating the data entries: (1) some (53/1498, 3.54%) came from latitude and longitude coordinates outside of the United States and its territories; (2) a little more than one-fifth (350/1498, 23.36%) were completed in record time (eg, 9 min vs an estimated completion time of 30 min); (3) some (355/1498, 23.69%) contained names or email addresses with unusual handles, unconventional characters, excessive numbers, or uncommon symbols (eg, a987quaiigi@yahoo.com and Â2xylzggf@gmail.com); and (4) a few (18/1498, 1.2%) included a US telephone number with an incorrect digit count (>10 digits or <10 digits). Upon further investigation, we noticed that responses to certain survey items were either nonsensical, highly improbable, or were incongruent with a response to a related item. For example, in an open-text item asking, “How did you hear about PrEP?” some respondents provided nonsensical answers, such as “Jsjsjd,” or an illogical response, such as “Yes” (27/1498, 1.8%). A highly improbable response pattern was observed when some entries (17/1498, 1.13%) selected all options for HIV behavioral risk factors (eg, “I had unprotected vaginal sex with a male partner [vaginal sex without using a condom] in the past 6 months,” “I had unprotected anal sex with a male partner [anal sex without using a condom] in the past 6 months,” “I had sex in exchange for something of value [such as food, shelter, money, or drugs] in the past 6 months,” “I have taken drugs by injection with a needle [such as heroin, cocaine, amphetamines, or steroids; not including anything taken under a doctor’s order] in the past 6 months,” and “I have been diagnosed with an STI in the past 6 months [STIs include chlamydia, gonorrhea, syphilis, human papilloma virus (HPV and warts) and herpes simplex virus]”). Selecting all options was considered highly unlikely.

After evaluating the influx of responses and noting the aforementioned patterns, we decided to call the first 20 people who met the eligibility criteria, consented to participate, and completed the study survey and whose responses showed no signs of fraudulent activity. The goal was to verify whether the provided telephone numbers were valid and belonged to the respondents. The majority of telephone numbers called (16/20, 80%) were linked to a person, company, or business not associated with the individual listed in the survey entry or were disconnected or no longer in service. Only 4 (20%) of the 20 telephone numbers were valid and belonged to the individual who completed the survey. In summary, our evaluation of data entries in relation to the limitations of the survey-based fraud prevention safeguard options highlighted the need to implement additional fraud deterrence and detection procedures to help ensure that valid data were being collected from legitimate research participants.

### Application of the Second Level of Fraud Detection: Lesson 2

We made a number of changes before resuming the study. First, we retained and implemented the original 4 safeguard options provided by Qualtrics. In addition to the existing CAPTCHA, which was placed before the first question of the eligibility screener, we added a CAPTCHA before the first question of the study survey to further deter bots. We also decided to manually evaluate all completed eligibility screeners (with the associated study survey) for duplicate records (ie, >1 entry) [38] by assessing whether the same IP address, telephone number, or email address appeared across multiple entries. If >3 entries were found to have the same IP address, we retained the first 3 and removed the rest. If multiple entries were found to have the same telephone number or email address, we kept only the first entry and removed all others. On the basis of prior research and our initial findings, we created and implemented 7 additional data validity benchmarks for fraud detection. Each benchmark included a predetermined decision rule that was created a priori. These benchmarks, along with their corresponding decision rules, are described in Table 1 and were implemented alongside the 4 original Qualtrics safeguard options.

After duplicates were removed, each screener and study survey entry was evaluated by applying the 7 fraud detection benchmarks presented in Table 1. First, any entry with latitude and longitude coordinates outside of the United States was labeled as fraudulent and excluded (ie, ineligible and not enrolled). Any entry that had a completion time of ≤10 minutes was also labeled as fraudulent and not enrolled. All remaining entries with latitude and longitude coordinates within the United States and a completion time of >10 minutes were further evaluated using the 5 remaining benchmarks (items 3-7 in Table 1). These 5 benchmarks evaluated whether the self-reported telephone number contained 10 digits in the United States format (ie, xxx-xxx-xxxx); the self-reported email address was correctly formatted and deemed valid using a third-party email validation tool or was associated with a Facebook profile; the responses to certain survey questions (eg, HIV behavioral risk factors or open-ended survey questions) were nonsensical or highly improbable (ie, selecting having engaged in all 5 options for HIV behavioral risk factors in the 6 months before study participation: unprotected vaginal sex with a male partner, unprotected anal sex with a male partner, injection drug use, sex exchange, and diagnosed with an STI); the self-reported responses to the items about zip code and residential city matched; and name, email address, or open-ended text responses contained nonstandard characters or symbols not commonly used in the United States (eg, Â). To be considered a highly probable respondent with valid data, individuals had to pass all 5 fraud detection benchmarks (items 3-7 in Table 1). Respondents who passed ≤3 were deemed fraudulent (ie, ineligible and not enrolled). Those who passed at least 4 benchmarks were marked for further review. A team member then contacted these individuals via telephone or email to ask 2 questions about the personal information provided in the screener or study survey (eg, current age, birth month, zip code, city of residence, or relationship status). Individuals who could not be reached or provided incorrect answers were categorized as “fraudulent” and not enrolled. The goal of these procedures was to identify additional fraudulent entries to improve the integrity of the data collected.

**Table 1:** Description of the level 2 fraud detection protocol, which included 7 data validation benchmarks and corresponding decision rules used to identify additional fraudulent survey respondent entries^a^.

Data used for benchmark	Benchmark rule	Data source	Decision rule
1. Latitude and longitude coordinates of IP address	Location must be within the United States	Eligibility screener	If individual does not pass, labeled as fraudulent; no longer reviewed
2. Start and finish time stamps	Completion time must exceed 10 minutes	Eligibility screener and study survey	If individual does not pass, labeled as fraudulent; no longer reviewed
3. Telephone number	Digits must be provided in the correct United States format (xxx-xxx-xxxx)	Eligibility screener	If not in United States format, labeled for further review
4. Email address	Email address must be valid when verified online through a third party (eg, s223456h@yahoo.com or have altering letters and numbers – 12s2n4n5P3@gmail.com [28,29])	Eligibility screener	If not in correct format or fake email address, labeled for further review
5. Nonsensical or highly improbable responses to survey questions	Selected all 5 options for HIV behavioral risk factors in the screener or provided irregular responses to open-ended survey questions	Eligibility screener and study survey	If all 5 options for HIV behavioral risk factors are selected, labeled for further review; if nonsensical or highly improbable responses have been provided to open-ended questions, labeled for further review
6. Matched responses: zip code and city	Provided a zip code that did not match their self-reported residential city	Eligibility screener	If self-reported zip code and city do not match, labeled for further review
7. Nonstandard characters or symbols	Name, email address, or open-ended text responses containing nonstandard characters or symbols not commonly used in the United States (eg, Â and Didnâ€™t)	Eligibility screener and study survey	If nonstandard characters or symbols identified, labeled for further review

^a^Respondents were first evaluated against items 1 and 2 of the 7 data validation benchmarks. Those who did not meet these criteria were labeled as “fraudulent.” Respondents who passed were then evaluated against the remaining 5 benchmark items (items 3-7) and labeled based on the number of criteria met: “pass” (met all 5 criteria), “further review” (met 4 criteria), and “fraudulent” (met ≤3 criteria).

### Ethical Considerations

The procedures associated with the primary study, including ethics approval and oversight, were approved by the institutional review board at the University of Hawaiʻi at Mānoa (IRB 2020-00030). A certificate of confidentiality for human participant research was obtained from the National Institutes of Health to help keep participant data private. Informed electronic consent was obtained from all participants included in the study; each respondent was required to individually provide consent electronically before taking the eligibility screener. Upon study completion, all documents containing identifying information were deidentified and coded with a unique study number. As approved by the ethics committee, enrolled participants (ie, individuals who provided consent, met the eligibility criteria, completed at least 80% of the study survey, and passed all fraud deterrence and detection protocols) were emailed a US $25 electronic gift card for taking part in the study.

## Results

### Consent, Eligibility Criteria, and Level 1 Fraud Deterrent Protocols

**Figure 1:**
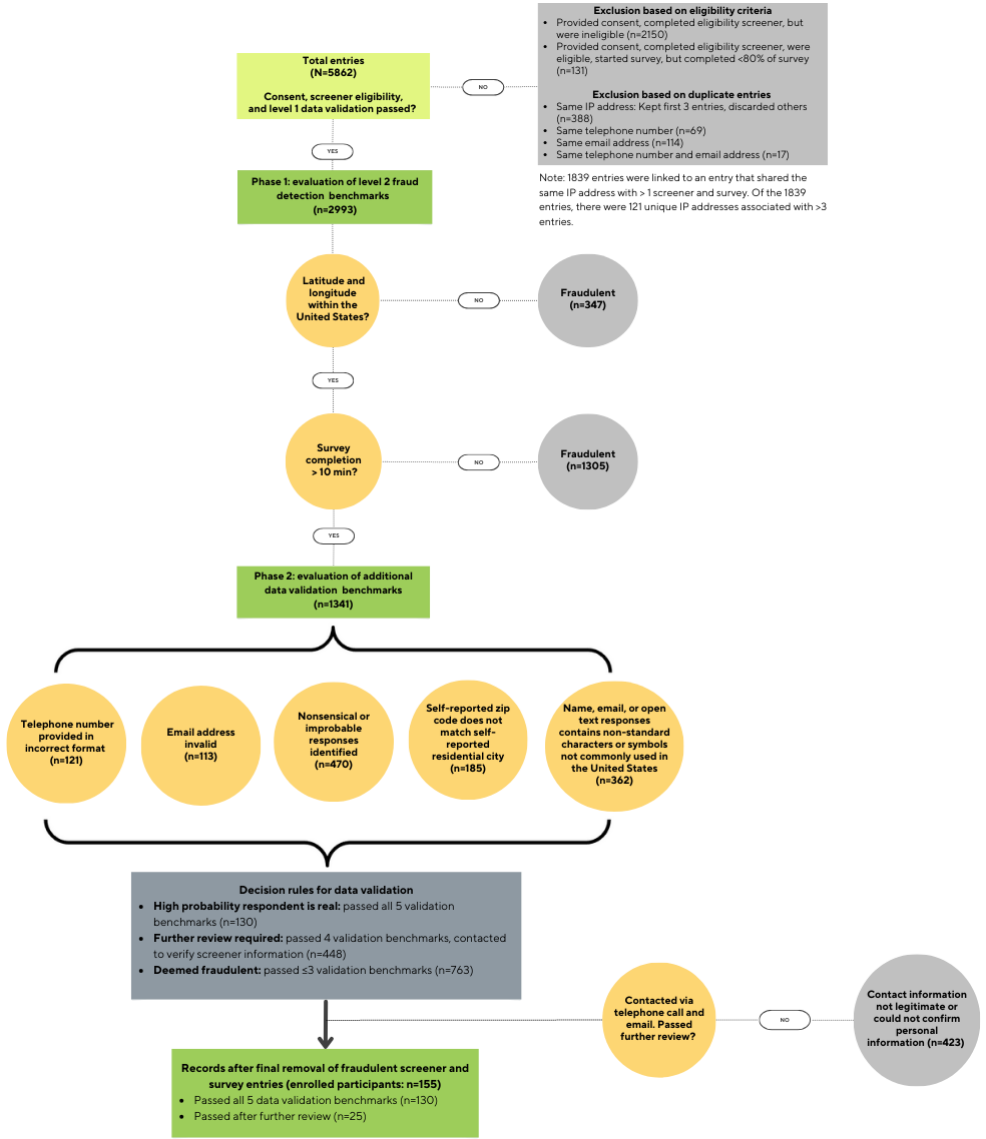
Final participant enrollment after implementing the 4 fraud deterrent safeguards (provided by Qualtrics) and the 7 fraud detection benchmarks.

Figure 1 presents the number of entries removed by implementing the 4 fraud deterrent safeguards provided by Qualtrics and the 7 fraud detection benchmarks. There were 5862 entries representing those who clicked the study link and provided consent. First, entries were removed if they did not pass the study eligibility criteria (ie, ineligible; 2150/5862, 36.68%). Entries were also removed if the respondents provided consent and were eligible but completed <80% of the study survey (131/5862, 2.23%). Second, duplicate entries (ie, entries with the same IP address and same telephone number or email address; 588/5862, 10.03%) were removed. With respect to duplicate IP address, there were 1839 entries that had the same IP address as at least 1 other screener and survey. Among these 1839 entries, 121 unique IP addresses were linked to an entry that shared the same IP address with ≥4 screeners and surveys. Following the data validation protocol for entries with the same IP address (ie, retaining the first 3 entries with the same IP address and discarding additional ones), 388 (21.1%) of the 1839 entries were removed. In sum, there were 2993 respondents who provided consent, met the eligibility criteria, and passed level 1 fraud deterrent safeguards.

### Level 2 Fraud Detection

Seven additional fraud detection benchmarks were implemented, resulting in the removal of additional entries. Of the 2993 entries that remained, 347 (11.59%) with latitude and longitude coordinates outside of the United States were removed, while 1305 (43.6%) were removed for having a survey completion time of <10 minutes. Among the remaining 1341 entries, implementing the 5 remaining fraud detection benchmarks led to the exclusion of 56.9% (763/1341) of the entries that met <4 of the benchmarks. Only 130 (9.7%) of the 1341 respondents passed all 5 data validation benchmarks and were enrolled in the study. A total of 448 respondents were contacted via telephone or email for further legitimacy review, of whom 25 (5.6%) were successfully reached and verified as legitimate.

### Enrolled Participants

After removing entries based on the fraud deterrence and detection protocols, 155 Black cisgender women met all study criteria, including providing consent, meeting eligibility requirements, completing at least 80% of the study survey, and passing all fraud deterrence and detection protocols, and were enrolled in the study.

## Discussion

### Principal Findings

This study, which focused on Black cisgender women, emphasizes the importance of implementing effective fraud deterrence and detection procedures in incentivized online research. Our findings, stemming from the lessons learned, highlight that solely relying on fraud deterrence features offered by online survey programs (eg, Qualtrics) is insufficient to ensure the collection of valid data from legitimate research participants. We demonstrate and argue that when conducting incentivized online research, additional fraud deterrence and detection protocols are needed to uphold data integrity and research rigor. These protocols should be implemented before the start of recruitment and enrollment, and should be adjusted as needed throughout the process. Of the 5862 entries received for the study, only 155 (2.64%) provided consent, were eligible, and met fraud detection and deterrent criteria. A large proportion of the entries (2838/5862, 48.41%) were deemed fraudulent. Through reflection on the protocols used in this online study, we offer suggestions and ideas for researchers to consider using to help deter and detect fraudulent data entries, with the objective of helping to increase the likelihood of collecting valid data from genuine participants.

### Leveraging Available Features in Survey Software Programs to Deter Bots

Effectively identifying and managing bots is important to ensure the accuracy, reliability, and integrity of collected data. We recommend using all available features provided by the survey software while also being aware of their limitations to deter fraud. In this online study, we used the CAPTCHA verification, prevent indexing, prevent ballot box stuffing (ie, duplicate entries), and bot detection features offered by Qualtrics. We recommend leveraging the CAPTCHA feature because it can be used more than once within an online survey. Although 1 prior study [24] found the CAPTCHA feature to be insufficient in dissuading fraudulent responses, the authors did not provide details about how often it was actually used in their survey. In this study, we strategically placed a CAPTCHA before the first question in the eligibility screener as well as before the first question in the study survey. Strategically placing a CAPTCHA before the initial question in both the eligibility screener and the study survey potentially aided in the identification and elimination of additional fraudulent entries that might have bypassed the initial CAPTCHA. This assertion is supported by the discovery of participants (n=131) who provided consent and met the eligibility criteria but did not start the survey (Figure 1). The strategic placement of CAPTCHAs provides an additional layer of security against automated bot submissions and may help reduce the likelihood of receiving invalid responses.

The “prevent ballot box stuffing” option uses cookies to prevent multiple survey entries but does not fully prevent duplicate entries based on IP address or self-reported information, such as name, email address, and telephone number. Although prior studies have used this fraud detection feature, its limitations were not acknowledged or discussed [28,37]. Our evaluation suggests that respondents, in an attempt to identify which responses would qualify them for the study, may have repeatedly taken the eligibility screener using different internet browsers, as evidenced by the high number of duplicate entries from the same IP addresses (n=1839). When we discovered this, we manually evaluated screener entries to label and assess which ones came from the same IP address. We then applied the same process to evaluate screener entries that contained the same email addresses and telephone numbers, given the limitations of the prevent ballot box stuffing option. The implementation of these evaluation methods was valuable in discerning whether entries originated from the same entity. Our experience and the relevant lessons learned underscore the importance of understanding the limitations of fraud deterrence features in online survey software.

It is important to acknowledge that the 4 fraud deterrence features (CAPTCHA verification, prevent indexing, prevent ballot box stuffing, and bot detection) were implemented in the Qualtrics-hosted study survey between December 2020 and January 2022. Since then, Qualtrics may have updated or enhanced these features. Furthermore, Qualtrics offers 2 other fraud deterrence features that were not used in this study: Security Scan Monitor and RelevantID. Security Scan Monitor prevents email scanning software from inadvertently starting a survey session when a survey link is included in the email. This feature applies to all links, regardless of whether they were distributed via Qualtrics or a third-party system (ie, any software or platform not directly associated with Qualtrics, such as marketing automation software). RelevantID analyzes respondent metadata to determine the likelihood of multiple survey attempts by the same respondent by examining browser, operating system, and location details. Future surveys that are hosted on Qualtrics ought to consider using Security Scan Monitor and RelevantID, depending on the needs of the study. We also recommend that researchers refrain from exclusively relying on the fraud deterrence features provided by Qualtrics (or other survey software). Our lessons learned indicate that using a combination of fraud deterrent and fraud detection procedures will help provide a more robust defense against the collection of invalid data in online survey studies.

### Enhancing Fraud Detection

Every online survey study is vulnerable to fraudulent entries. As such, the best approach is to identify potential vulnerabilities and implement strategies to prevent their exploitation before starting recruitment and data collection [25]. Initially, researchers should create and implement a protocol that incorporates both fraud deterrent and fraud detection strategies. Drawing from our experiences with this online survey study, we found that a combined approach of fraud deterrent and fraud detection worked best to identify fraudulent entries. Specifically, we—as well as prior research—evaluated the time taken to complete the eligibility screener and study survey [21,24,28,29,36], contacted respondents via telephone or email to verify their identity [36,39], and used matched survey questions to identify nonsensical or highly improbable responses [21,24,29].

Predetermining an anticipated time range for survey completion adds an additional option to help detect fraud. For instance, data collected about survey completion time can be used to decipher whether a “participant” was likely to have completed it. Establishing a priori criteria for anticipated eligibility screener and study completion time can aid in fraud detection. Before launching this study, we used the automated survey completion time estimate provided by Qualtrics, which was 53 minutes. However, while this estimate accounts for survey flow, it does not account for skip logic or display logic. To address this, we manually tested survey completion times across different response paths. Our completion times ranged between 18 and 30 minutes. On the basis of these findings, we determined that it would be highly unlikely for an individual to complete the eligibility screener and associated study survey in <10 minutes. Therefore, we flagged all entries that completed the screener and survey in <10 minutes and considered them to represent automated, nonhuman involvement or rushed responses. We recommend that researchers consider implementing a similar strategy by predetermining a time range for survey completion to help detect fraudulent or rushed survey entries.

Similar to prior research [24,28], we verified respondents’ telephone numbers and email addresses. The verification of contact information is important because respondents can easily create multiple email addresses or use Google Voice numbers to complete the survey multiple times. Third-party services that validate email addresses and Facebook were used to verify email addresses, while telephone numbers were validated through direct telephone calls to confirm legitimacy and ownership. Respondents labeled for “further review” were contacted via telephone or email and asked 2 questions about the personal information provided in the screener or study survey (eg, current age, birth month, zip code, city of residence, and relationship status). Although resource intensive, verifying phone numbers and emails helped to differentiate between legitimate and fraudulent entries, offering confidence in the authenticity of participant details and their survey data.

A notable concern with manual checks and using third-party verification services is the associated cost and time commitment. These expenses can be significant, especially for research projects with limited budgets. The time-consuming nature of manual verification of telephone numbers becomes apparent when handling a high volume of entries within a short time frame. Calling respondents individually can lead to delays and logistical challenges, impacting the efficiency of the research process. To deter fraudulent behavior, Ballard et al [28] sent an SMS text message to suspicious respondents stating, “You recently completed a survey for a health study online. However, we detected that your survey entry was fraudulent. If you think this is a mistake, please contact us.” If participants did not respond, the authors considered the survey entry invalid [28]. The authors noted that they did not receive any responses categorized as “fraudulent” [28]. In summary, researchers must weigh the benefits of enhanced participant validation against practical constraints, such as cost, time, and personnel involvement, when considering the adoption of email and telephone verification protocols.

We also incorporated a matched response approach using 2 survey questions to enhance our fraud detection protocol. Respondents’ self-reported zip code had to match their self-reported residential city; otherwise, they were flagged for possible fraud. Matching responses to different survey questions can help identify potential fraudulent entries. We recommend that researchers consider using at least 1, if not 2, matched responses to preselected survey questions. For example, asking for a respondent’s current age at the beginning of an eligibility screener and then requesting their birth month and year—either toward the end of the screener or in the study survey—may help reveal discrepancies [34,36,37]. Alternatively, a survey could ask for a respondent’s age range at the beginning and their specific age toward the end.

### Additional Strategies and Recommendations for Fraud Deterrence and Detection

There may be other options that work best for online survey studies that can be used to discourage fraudulent behavior. Pratt-Chapman et al [24] recommend including a check box for participants to acknowledge that responses from ineligible respondents or multiple entries from the same respondent will disqualify them from receiving financial incentives. The authors also suggest indicating the investigators’ right to confirm eligibility by telephone (or other means) to aid in identifying bots and eliminating duplicate entries [24]. In addition, when requests for payment are received from respondents whose entries are identified as fraudulent, Dewitt et al [29] recommend informing these respondents that there was a concern about their survey entry and asking them to call a 1-800 study line and leave a callback number for verification. The authors applied this approach to all suspicious entries and found that none resulted in return calls [29]. Another option is to contact participants via telephone or email to set up a Zoom meeting to determine their legitimacy [39] before inviting them to complete the study survey. During this meeting, participants could be asked to upload or show evidence to prove their identity or to verify they had met some of the inclusion criteria; for example, a study recruiting active US military service members could require potential participants to show their military ID as proof of service. The methods described here offer additional strategies for deterring and detecting fraudulent entries in online research studies.

While manual checks can be a viable option for online research studies with limited resources, more financially robust studies can deploy advanced fraud deterrence and detection strategies. One such option is incorporating email and telephone verification features that require respondents to validate their contact information by receiving and verifying a code. Guest et al [36] required respondents to submit their mobile phone number during the eligibility screener to receive a 3-digit verification code via SMS text message. After receiving the 3-digit code, respondents were required to enter the code in the eligibility screener survey to validate their mobile phone number for the study [36]. Those who failed to input the code were unable to continue with the eligibility screener [36].

Another recommendation for online eligibility screening is the use of automated electronic algorithms that allow researchers to create parameters and decision rules for inclusion criteria. Automated electronic algorithms also enable online eligibility screeners to restrict entries based on IP address (ie, blocking multiple entries from the same IP address or requiring a US-based IP address), completion time, and allowable responses that align with the inclusion criteria (eg, current age must be at least 18 years) [37]. These algorithms enhance fraud deterrent strategies while streamlining the eligibility screening process. However, implementing such techniques requires adequate financial and computational resources (eg, a coder or a web developer) tailored to the study’s specific needs. It should be noted that implementing these procedures results in a major reduction in the study sample size. This is often the case when researchers opt to prioritize data integrity to ensure that the findings are representative of real people.

Overall, ongoing efforts are needed to refine and optimize fraud deterrence and detection protocols to maintain research rigor and improve the collection of valid data from legitimate research participants. This study uniquely contributes to existing related literature because it is one of the first to evaluate, describe lessons learned, and offer insights into fraud deterrence and detection protocols used for an incentivized online survey study with adult Black cisgender women. Most online research studies that have evaluated the use of fraud deterrence and detection methods have focused on samples of sexual minority men and male couples [11,12,23,34,37]. Further research is needed to evaluate the application and effectiveness of fraud deterrence and detection methods in online studies with diverse populations. It remains unknown whether distinct demographic groups require unique fraud deterrence and detection procedures. We encourage researchers to evaluate and publish findings stemming from their use of fraud deterrence and detection methods to help advance the rigor of online research studies.

### Limitations

The limitations of this study are important to consider in light of the lessons learned and the recommendations provided. Despite the changes we made to enhance the fraud deterrence and detection methods used in this online survey study, it is possible that some participants provided false information during the verification attempts. In addition, reliance on telephone calls and emails for participant validation may introduce biases or errors due to participant nonresponsiveness or communication challenges. Participants may forget to respond to telephone calls or emails. As a result, there may be inaccuracies in the data. Although we used a third-party service for email validation, it is possible that some of the verified email addresses belonged to individuals other than the persons or entities completing the eligibility screener. Moreover, the evolving nature of fraud detection algorithms in online survey platforms such as Qualtrics may limit the long-term generalizability of recommendations based on research conducted within specific time frames. In addition, this study’s fraud detection and deterrent protocols were designed to recruit Black cisgender women, potentially limiting the generalizability of these protocols to other populations. Future research should aim to use these protocols and provide feedback regarding whether these protocols improved their data integrity by improving their ability to identify bots and imposter participants during recruitment and enrollment. The findings would help to refine and strengthen fraud detection and deterrent protocols used to recruit diverse populations for online studies. It is important to acknowledge that the use of fraud deterrence and detection methods does not guarantee the complete elimination of all fraudulent entries; however, their use and evaluation help to enhance the confidence that valid data are being collected from verified participants, thereby contributing to the rigor and integrity of online research studies.

### Conclusions

Effectively identifying fraudulent responses in web-based surveys is an ongoing challenge. With the increasing shift toward web-based research and online recruitment, the threat of fraudulent participation poses a real challenge to data validity. Protocols for identifying fraudulent survey entries and verifying and validating potential study participants should be considered for all internet-based studies. Researchers conducting online studies with Black cisgender women must actively share their experiences in deterring and detecting fraud to help contribute to the rigor of best practices and maintaining the validity of data and associated findings. The lessons learned and recommendations offered from the experiences of conducting this online study, which recruited and enrolled a study sample of Black cisgender women, highlight two important take-home points: (1) develop a thorough fraud deterrent and fraud detection plan to implement before study launch; and (2) monitor and evaluate how well these methods are working while data are being collected, as well as once data collection has ended. We encourage researchers to leverage all resources they may have at their disposal, given the number of different fraud deterrence and detection options that exist. This study—which emphasizes the importance of the aforementioned take-home points—used a combination of fraud deterrence and detection methods to identify a large number of fraudulent entries that would have otherwise been included in the data.

## Appendix

Multimedia Appendix 1 STROBE Checklist.

## Abbreviations

STI: sexually transmitted infection
STROBE: Strengthening the Reporting of Observational Studies in Epidemiology

## Data Availability

The datasets generated and analyzed during this study are not currently publicly available because the primary data are still being analyzed. Once primary analyses are complete, the study data will be available from the corresponding author on reasonable request.
